# Genome Wide Identification and Functional Prediction of Long Non-Coding RNAs Responsive to *Sclerotinia sclerotiorum* Infection in *Brassica napus*

**DOI:** 10.1371/journal.pone.0158784

**Published:** 2016-07-07

**Authors:** Raj Kumar Joshi, Swati Megha, Urmila Basu, Muhammad H. Rahman, Nat N. V. Kav

**Affiliations:** 1 Department of Agricultural Food and Nutritional Science, University of Alberta, Edmonton, Alberta, T6G 2P5, Canada; 2 Centre of Biotechnology, Siksha O Anusandhan University, Bhubaneswar-751003, India; Washington State University, UNITED STATES

## Abstract

Sclerotinia stem rot caused by *Sclerotinia sclerotiorum* affects canola production worldwide. Emerging evidence suggests that long non-coding RNAs (lncRNAs) play important roles in the regulation of gene expression in plants, in response to both abiotic and biotic stress. So far, identification of lncRNAs has been limited to a few model plant species, and their roles in mediating responses to biotic stresses are yet to be characterized in *Brassica napus*. The present study reports the identification of novel lncRNAs responsive to *S*. *sclerotiorum* infection in *B*. *napus* at two time points after infection (24 hpi and 48 hpi) using a stranded RNA-Sequencing technique and a detection pipeline for lncRNAs. Of the total 3,181 lncRNA candidates, 2,821 lncRNAs were intergenic, 111 were natural antisense transcripts, 76 possessed exonic overlap with the reference coding transcripts while the remaining 173 represented novel lnc- isoforms. Forty one lncRNAs were identified as the precursors for microRNAs (miRNAs) including miR156, miR169 and miR394, with significant roles in mediating plant responses to fungal phytopathogens. A total of 931 differentially expressed lncRNAs were identified in response to *S*. *sclerotiorum* infection and the expression of 12 such lncRNAs was further validated using qRT-PCR. *B*. *napus* antisense lncRNA, TCONS_00000966, having 90% overlap with a plant defensin gene, showed significant induction at both infection stages, suggesting its involvement in the transcriptional regulation of defense responsive genes under *S*. *sclerotiorum* infection. Additionally, nine lncRNAs showed overlap with cis-regulatory regions of differentially expressed genes of *B*. *napus*. Quantitative RT-PCR verification of a set of *S*. *sclerotiorum* responsive sense/antisense transcript pairs revealed contrasting expression patterns, supporting the hypothesis that steric clashes of transcriptional machinery may lead to inactivation of sense promoter. Our findings highlight the potential contributions of lncRNAs in regulating expression of plant genes that respond to biotic stress.

## Introduction

Whole genome RNA sequencing (RNA-Seq), tilling arrays and large-scale cDNA cloning studies have revealed that transcription of eukaryotic genes is highly complex [1–2]. Although 90% of the eukaryotic genome is transcribed, only 1–2% has the capacity to encode proteins, suggesting that a large fraction of eukaryotic transcripts represent non-coding RNAs (ncRNAs) [3–4]. The specific functions of most of the ncRNAs has not yet been fully characterized, but recent evidence from plants and animals supports a crucial role of these ncRNAs in the regulation of a wide range of biological processes including plant responses to biotic and abiotic stresses [5–8]. Based on their length, ncRNAs are randomly grouped into short (< 200 nucleotides) and lncRNAs (> 200 nucleotides) [6]. The importance of small ncRNAs including microRNAs (miRNAs), short interfering RNAs (siRNAs), natural antisense siRNAs (nat-siRNAs), piwi interacting RNAs (piRNAs) and trans acting siRNAs (tasi-RNAs) in transcriptional and post-transcriptional regulation of gene expression in plants have been well documented [9–11]. Although, the molecular mechanisms governing the lncRNA-mediated gene regulation is still poorly understood, increasing number of studies suggest their involvement in the regulation of biological processes in plants [12–14].

LncRNAs are broadly classified as long intronic ncRNAs, long intergenic ncRNAs or natural antisense transcripts (NATs) based on their location [14]. They can be in either sense or antisense orientation in relation to the neighbouring protein-coding transcript and may overlap with exons or introns of protein coding genes [7]. However, systematic identification and classification of lncRNAs have been limited to a few model plant species such as *Arabidopsis thaliana*, rice and maize. In *A*. *thaliana*, 6,480 lncRNAs and 37, 238 NATs have been identified and classified using genome wide tilling array and RNA-Seq [15]. Similarly, in rice, 3,819 cis-NATs have been identified, among which 503 cis-NATs seem to be associated with specific biological functions such as growth and abiotic stress tolerance [16]. An exploitation of the expressed sequence tags (EST) databases, whole genome sequence annotation and RNA-Seq datasets from 30 different experiments led to the identification of 20,163 lncRNAs in maize [17]. Both strand-specific and non-directional sequencing in rice and maize identified 22,334 lncRNAs and 6,673 pairs of NATs and genome-wide association studies highlighted their involvement in developmental agricultural traits [18]. In another study, 8,449 drought responsive transcripts were analyzed with the coding potential calculator to identify 664 drought responsive lncRNAs in maize [19]. More recently, 2,542 lncRNA candidates have been identified from *Populus trichocarpa*, 504 of which were found to be drought responsive [20]. Interestingly, a number of lncRNAs serve as precursors for small RNAs including small nuclear RNAs (snRNAs), small nucleolar RNAs (snoRNAs) and miRNAs to regulate their functions [21]. Moreover, a number of lncRNAs act as endogenous target mimics for miRNAs and inhibit their functions, thus playing asignificant role in the regulation of plant growth and development [20–22].

Although a large number of lncRNAs have been identified in model plants, only a few of them have been functionallycharacterized. For instance, *Enod40*, a lncRNA from *Medicago truncatula* acts as molecular cargo for *M*. *truncatula* RNA binding protein 1 (MtRBP1) by relocalizing the protein from nuclear speckles into cytoplasmic granules during nodulation [23]. Induced by Phosphate Starvation 1(*IPS1*) lncRNA from *A*. *thaliana* modulates phosphate uptake by acting as a target mimic of miR399 and inhibiting its cleavage activity [24]. Two lncRNAs, COOLAIR and COLDAIR, transcribed from the *FLOWERING LOCUS C (FLC)* gene of *A*. *thaliana* play important rolesin the regulation of cold stress response [12–13]. COOLAIR is induced during vernalization and transcribed in an antisense orientation to *FLC* but is not essential for *FLC* repression [12]. In contrast, COLDAIR is transcribed from the first intron in the sense orientation relative to *FLC* and interacts with CURLY LEAF (CLF) of polycomb receptor complex 2 (PRC2) during vernalization [13]. It has been observed that the vernalization process is compromised in COLDAIR knockdown lines, suggestingits involvement in the regulation of FLC expression [13]. Additional lncRNAs such as *CsM10* from cucumber [25] and *zm401* from maize [26] have been found to be expressed in developing male gametophyte. A couple of recent studies have demonstrated an involvement of lncRNAs in biotic stress responses. For instance, 125 lncRNAs have been identified from wheat, which are responsive to powdery mildew infection [27]. These lncRNAs were not conserved among plant species and exhibited high tissue specific expression. In addition, 159 novel intergenic transcriptionally active regions (TARs) responsive to *Fusarium oxysporum* were characterized from *A*. *thaliana* [28]. Functional characterization of lncTARs through RNA-interference knockdown demonstrated direct involvement of five lncRNAs in disease development [28]. All of these reports provide evidence in support of the prominent roles of lncRNAs in the regulation of plant growth, development and stress responses.

*S*. *sclerotiorum* is a necrotrophic, non-host specific phytopathogen with over 400 different potential hosts [29]. In a wide range of host plants includingthe agriculturally important oil seed crop canola (*B*. *napus*), the fungus causes Sclerotinia stem rot [30]. Substantial yield losses from the outbreaks of Sclerotinia stem rot have been reported worldwide including in Australia, Europe and North America [31–32]. Comprehensive genome and proteome and analyses of defense signal transduction pathways of both the host plant and the pathogen have provided detailed insights about the molecular mechanisms underlying disease progression in the *B*. *napus-S*. *sclerotiorum* pathosystem [33–35]. However, the roles of lncRNAs in the regulation of antifungal defense network in *B*. *napus*, specifically in response to *S*. *sclerotiorum*, are still unknown. In the present study, we used a high throughput stranded RNA-Seq based approach, to elucidate lncRNA expression profiles in *B*. *napus* subsequent to infection with *S*. *sclerotiorum*. We have identified numerous *S*. *sclerotiorum* responsive lncRNAs which can be classified into different categories and may play key roles during the *B*. *napus-S*. *sclerotiorum* interaction. To our knowledge, this is the first report identifying the differentially expressed (DE) lncRNAs in this pathosystem and our findings may have long-term implications for the development of novel disease resistant canola lines.

## Materials and Methods

### Plant material, pathogen inoculation and RNA sequencing

A strain of *S*. *sclerotiorum* was kindly provided by Dr. Stephen Strelkov (University of Alberta) and seeds of *B*. *napus* cv. DH12075 were kindly provided by Dr. Gerhard Rakow of Agriculture and Agri-Food Canada (AAFC). *B*. *napus* cv. DH12075 (susceptible to *Sclerotinia* stem rot) seeds were surface sterilized and sown in plastic trays containing Metro Mix 290 (Grace Horticultural products, Ajax, ON, Canada). Plants were grown in the greenhouse with a 16 h photoperiod (light intensity of 130 μ mol m^-2^ s^-1^) with a day (21±1°C)/ night (18±1°C) temperature cyclefor 20 daysand transferred to ahumidity chamber 24 h prior to inoculation. Mature sclerotia of a wild-type *S*. *sclerotiorum* collected from an infected canola plant was used as the source of infection. Sclerotia were sub-cultured on potato dextrose agar medium (PDA; Becton Dickinson, Columbia, MD) and incubated at room temperature (RT; 21 ± 2°C) for 3 days. Agar plugs (0.2 cm) were removed with a sterile cork borer from the leading edge of the mycelia and were sub-cultured on PDA agar plate, grown for an additional 3 days at RT and used for inoculation of plants. Mycelial plugs (5 mm) excised from the culture plates with actively growing fungal cultures were placed on the first and second true leaves of *B*. *napus* which were slightly wounded prior to exposure to the pathogen. Leaves of un-inoculated plants were wounded and inoculated with sterile PDA plugs excised from plates without fungal cultures and will be referred to as control plants. Pathogen inoculated and control plants were placed in a humidity chamber for 24 h, after which they were returned to the greenhouse. The entire leaf samples (both first and second true leaves) from control and inoculated plants were harvested across all replicates and flash frozen in liquid nitrogen for RNA isolation at 24 and 48 hours post-infection (hpi).

Total RNA was extracted from *B*. *napus* leaves using TRIzol reagent (Invitrogen, USA) and treated with DNase (Promega, Madison, WI) according to manufacturer’s instructions. The quality and quantity of total RNA was determined using NanoDrop ND-1000 spectophotometer (Thermo Scientific, Waltham, USA). Integrity of isolated RNA was determined on Agilent 2100 Bioanalyzer using Plant RNA Nano chip assay in accordance with manufacturer’s instructions (Agilent Technologies, Santa Clara, CA). RNA samples with 260/280nm ratio between 2.0 to 2.1, and RNA integrity number (RIN) greater than 8.0, were used for the analysis. Total RNA from 12 samples, six un-inoculated (three each for 24 hpi and 48 hpi) and six *S*. *sclerotiorum* infected (three each for 24 hpi and 48 hpi) were used to construct RNA-Seq libraries using the TruSeq stranded total RNA preparation kit with RiboZero plant (Illumina, San Diego CA) according to the manufacturer’s instructions. The sequencing of libraries was performed on Illumina HiSeq 2500 platform at the Beijing Genomics Institute (BGI Americas, San Diego, CA).

### Assembling RNA transcripts and lncRNA detection

**Fig 1:**
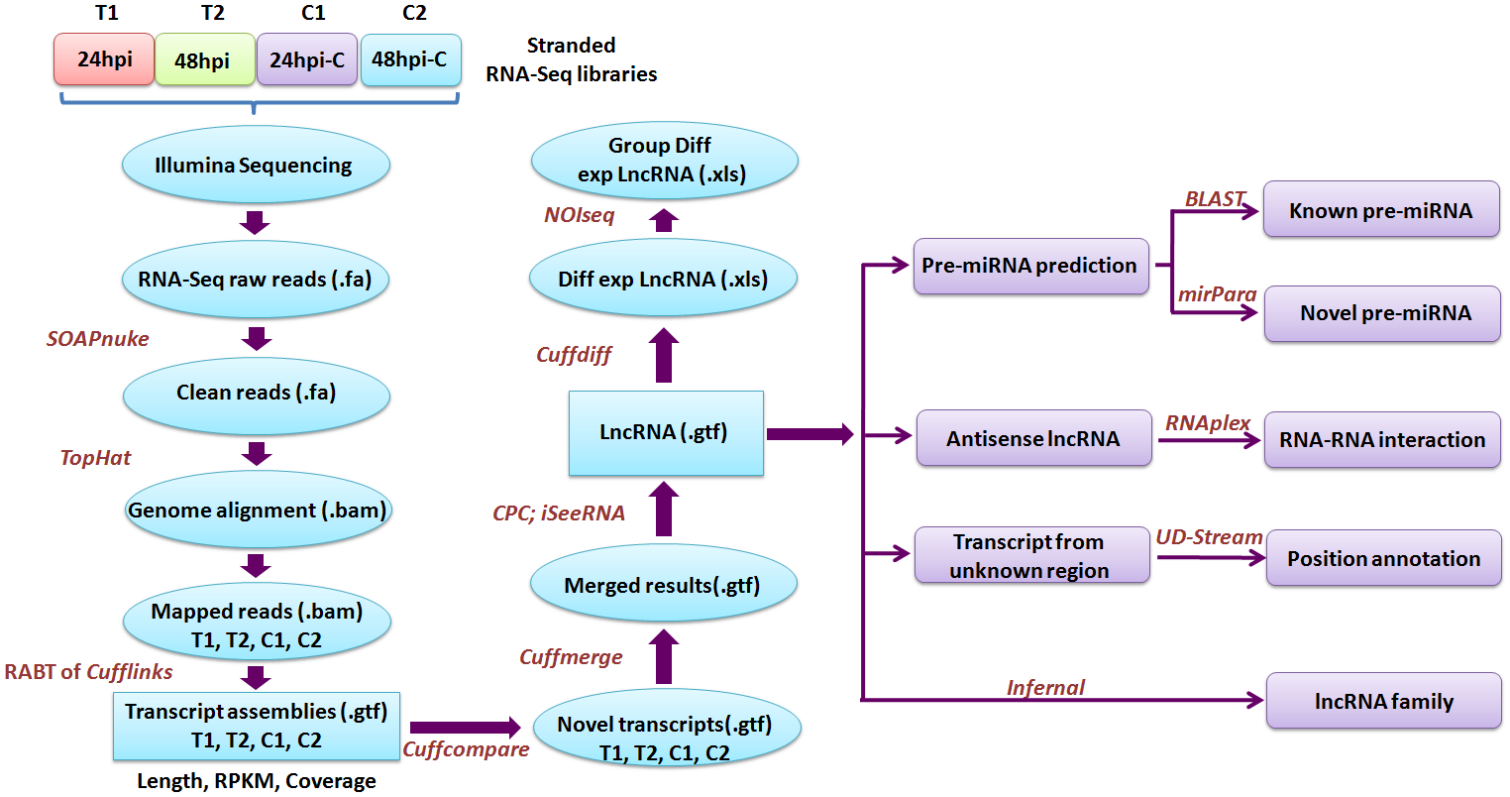
Pipeline for identification, annotation and classification of lncRNAs. Raw reads generated through stranded RNA-Seq were cleaned and mapped onto the reference genome of *B*. *napus*. Mapped reads were subsequently assembled by *Cufflinks*. A RABT assembly was performed with the reference gene annotation to compensate incompletely assembled transcripts. Novel set of transcripts were detected and categorized by utilizing *Cuffcompare*. *Cuffmerge* was used to merge the replicate assemblies and filter out transfrags. Low quality assemblies were filtered based on transcript length ≥ 200 nucleotides, open reading frames (ORFs) ≤ 300 nucleotides and RPKM ≥ 2.0. Finally, novel transcripts merged from different assemblies were evaluated using coding potential calculator (CPC) and iSeeRNA to identify transcripts with non-coding functions. INFERNAL was used to classify lncRNAs into various ncRNA families. miRNA precursors were identified using miRPara. Potential sense/antisense interactions were determined using RNAPlex. Intergenic lncRNAs were annotated as upstream or downstream lncRNAs using UD stream.

The basic approach used for transcript assembly and detection of lncRNAs is represented in Fig 1. Raw reads in the FASTAQ files were processed to remove adaptors, low-quality tags and contaminants. To remove the remaining rRNA reads, clean reads were mapped to the ribosomal RNA (rRNA) reference using soft read alignment software SOAPAligner/SOAP2 [36]. *B*. *napus* (AACC, 2n = 38) is an amphidiploid species which originated from interspecies crosses between *B*. *rapa* (AA, 2n = 20) and *B*. *oleracea* (CC, 2n = 18). As the complete sequence of the *B*. *napus* genome was not available at the time of this study, filtered reads were mapped to *B*. *rapa* (AA genome) [37] (http://www.brassicadb.org) and *B*. *oleracea* (CC genome) [38] (http://www.ocri-genomics.org/bolbase/) genomes respectively, using TopHat2 [39]. Also, the sequence reads were mapped to the *S*. *sclerotiorum* genome [40] (http://www.genome.jgi.doe.gov/Sclsc1/) and the mapped reads were removed from further analysis. Subsequently, the reads mapped onto the *B*. *rapa* (AA) and *B*. *oleracea* (CC) genomes were assembled by *Cufflinks* [41]. A reference Annotation Based Transcripts (RABT) assembly [42] was generated with the reference gene annotation, to compensate for incompletely assembled transcripts caused by read coverage gaps in the regions of reference gene. Novel transcripts were detected and categorized into different categories according to their position compared with reference genes by utilizing *Cuffcompare* [41]. Low quality assemblies were filtered according to transcript length ≥ 200 nucleotides, transcripts with open reading frames (ORFs) ≤ 300 nucleotides and an optimum RPKM (reads per kilobase transcriptome per million mapped) (RPKM ≥ 2.0) value. Cuffmerge was used to merge the replicate assemblies and filter out transfrags [41]. Finally, novel transcripts merged from different assemblies were evaluated using coding potential calculator (CPC) [43] and iSeeRNA [44] to predict transcript with non-coding functions. Using iSeeRNA, the unannotated transcripts were categorized into candidate categories such as ‘i’, ‘j’, ‘o’, ‘u’ and ‘x’, which may contain novel lncRNAs. The ‘i’ category may include lncRNAs within the introns of known genes, the ‘j’ category may contain alternative lncRNAs isoforms of known genes, the ‘u’ category may include the intergenic lncRNAs, the ‘o’ category may contain lncRNAs with exonic overlap with a known transcript, while the ‘x’ category may include exonic lncRNAs present on the opposite strand.

### Screening of differentially expressed lncRNAs

LncRNA sequence reads from control and pathogen infected libraries for 24 hpi and 48 hpi were normalized to FPKM (fragments per kilobase of transcript per million mapped reads) [41]. Differential expression test between the two comparisons was performed using Cuffdiff [41]. Fold changes for differentially expressed lncRNAs were calculated as: log_2_ ratio = log_2_ (FPKMinfected/ FPKMcontrol). False discovery rate (FDR) was determined after Benjamini-Hochberg correction [45] to identify false positive and false negatives. LncRNAs were considered DE, significant and pathogen responsive when log_2_ ratios of pathogen-inoculated samples compared to control were ≥2, and FDR adjusted *P* value (q-value) was ≤0.05. Furthermore, the DE lncRNAs between two time points were screened using NOISeq [46] by developing noise distribution models and Pearson’s correlation coefficients were calculated to determine the correlation of the DE lncRNAs between control and infected samples.

### LncRNA classification and functional prediction

Predicted lncRNAs were classified into different ncRNA families using INFERNAL, which categorizes ncRNAs and their conserved primary sequence and RNA secondary structure through the use of multiple sequence alignments (MSAs), consensus secondary structure annotation and covariance models (CMs) [47]. Potential miRNA precursors (pre-miRNAs) were predicted by subjecting the lncRNAs to BLAST search against the miRBase (www.mirbase.org) [48] and selecting hits with sequence coverage more than 90%. Subsequently, a support vector machine (SVM) based software mirPara [49] was used to detect novel miRNAs. All antisense lncRNA-mRNA duplexes exhibiting complementary base pairing were screened using RNAplex [50] to determine the potential antisense lncRNA-mRNA interaction. Finally, lncRNAs belonging to "unknown region" in former analyses were either classified as upstream or downstream, in order to determine their involvement in transcriptional regulation of corresponding genes.

### Quantitative real time polymerase chain reaction (qRT-PCR) analysis

Twelve randomly selected, pathogen responsive lncRNAs and six corresponding sense transcripts from *B*. *napus* were validated using qRT-PCR. Sequences of primers used in qRT-PCR analyses are provided in S1 Table. All qRT-PCR analyses were performed on the StepOne Plus real time PCR system (Life Technologies, Burlington, ON, Canada) using FASTSYBR green mix from Kappa Biosystem (D Mark, Toronto, ON, Canada). RNA isolated from pathogen-inoculated and control samples were treated with RNAse-free DNase I. Total RNA (1μg) was reverse transcribed using random primers and SuperScript III reverse transcriptase (Invitrogen). Control reactions without reverse transcriptase were performed for each primer combination in order to ensure the absence of genomic DNA contamination. Cycle threshold (C_T_) values were determined for each sample based on three biological replicates each with two technical replicates. Constitutively expressed *UBC9* (Ubiquitin conjugating enzyme 9) gene was used as the reference. Relative expression of the target transcripts was calculated using the 2^−ΔΔCT^method [51].

## Results and Discussion

### *B*. *napus* disease symptoms in response to *S*. *sclerotiorum*

The progression of infection was monitored in the susceptible *B*. *napus* cv. DH12075 to correlate the host responses with pathogen invasion. Symptoms of leaf necrosis and pathogen invasion have been described in our previous study [52]. The results demonstrated that, the area of leaf necrosis and amount of fungal mycelia detectable in the host tissue increased from 24 hpi to 48 hpi and was consistent with the severity of the observed, macroscopic disease symptoms.

### Deep sequencing and identification of lncRNAs

Strand specific sequencing reveals antisense transcription activities and assists in the identification of novel lncRNAs. Recently, this approach has been utilized by plant biologists for analyses of transcriptomes and also towards characterization of a wide range of ncRNAs in different plant species [16, 20, 28]. To identify the lncRNAs involved in mediating responses to *S*. *sclerotiorum* infection of *B*. *napus*, a strand specific Illumina sequencing of transcripts from canola leaves at 24hpi and 48hpi was performed. A total of 317 million clean reads were obtained from the 12 libraries (S2 Table) and approximately 204 million reads (64.56%) were successfully mapped on to the reference genomes (AA genome of *B*. *rapa* and CC genome of *B*. *oleracea*). The RNA-Seq reads have been deposited at the sequence read archive (SRA) of NCBI (accession numbers SRX956927 to SRX956938). Among transcripts, 62,071 multi-exon transcripts were assembled and 43,714 of the assembled transcripts were completely annotated, 393 transcripts were partially annotated while the remaining 17,964 transcripts (S3 Table) were not annotated and are potentially novel in nature. The assembly of 17,964 transcripts was further categorized into initial categories of lncRNAs (Table 1). Among the 17,964 sequences, 12,542 transcripts were classified with classcode ‘u’, 4,127 transcripts were classified in the ‘j’ category, 716 transcripts represented the ‘x’ category while the remaining 579 were grouped under the ‘o’ category. Based on the FPKM ≥ 2.0 as cut off, we identified a total of 3,181 transcripts from the previous four categories as *B*. *napus* lncRNAs responsive to *S*. *sclerotiorum* (S4 Table) (Table 1). In total, 2,821lncRNAs were assigned classcode ‘u’ as they were located in the intergenic regions. The remaining lncRNAs had an exonic overlap with known genes. For instance, 111lncRNAs with classcode ‘x’ had exonic overlap with known genes in the opposite strand, while 76 lncRNAs with classcode ‘o’ had generic exonic overlap with the reference coding transcripts. Additionally, 173 lncRNAs categorized with classcode ‘j’ represented novel lnc isoforms with at least one splice junction shared with the reference genes (Table 1). Thus, we could predict and classify *B*. *napus* lncRNAs into four biotypes as per the categories laid down in the NONCODE ver. 4.0 database [53]. Earlier, this pipeline was applied to a mouse Krueppel-like factor 1 (*Klf1*) knockout single-end RNA-Seq dataset to identify 308 novel lncRNA candidates [54]. We could predict a higher number of lncRNAs in our current study using the same pipeline, perhaps due to the fact that paired end sequencing and strand specific sequencing of *B*. *napus* transcript improved the prediction accuracy of the lncRNA detection tool. This premise is also supported by a recent report where a genome wide analysis of lncRNAs using a strand specific paired-end RNA-Seq experiment identified 2,542 lncRNAs in *P*. *trichocarpa* [20]. These observations suggest that the use of high quality paired-end reads generated through strand specific deep sequencing can act in an unbiased way to capture maximum long non coding transcriptome.

**Table 1:** Categories of initial assemblies and classification of novel lncRNAs from *B*. *napus* post infection with *S*. *sclerotiorum*.

Class code	After initial assemblies		After final processing		Description
	Transcript no.	%	Transcript no.	%	
=	43,714	70.42	**-**	**-**	Complete match of intron chain
C	393	0.63	**-**	**-**	Contained by a reference transcript
J	4,127	6.64	173	5.43	Potentially novel isoform (Atleast one splice junction is shared with a reference transcript)
O	579	0.93	76	2.38	Generic exonic overlap with a reference transcript
U	12,542	20.20	2,821	88.68	Intergenic transcript
X	716	1.15	111	3.48	Exonic overlap with reference on the opposite strand
Total	62,071	100%	3,181	100%	

### Distribution of *B*. *napus* lncRNAs

**Fig 2:**
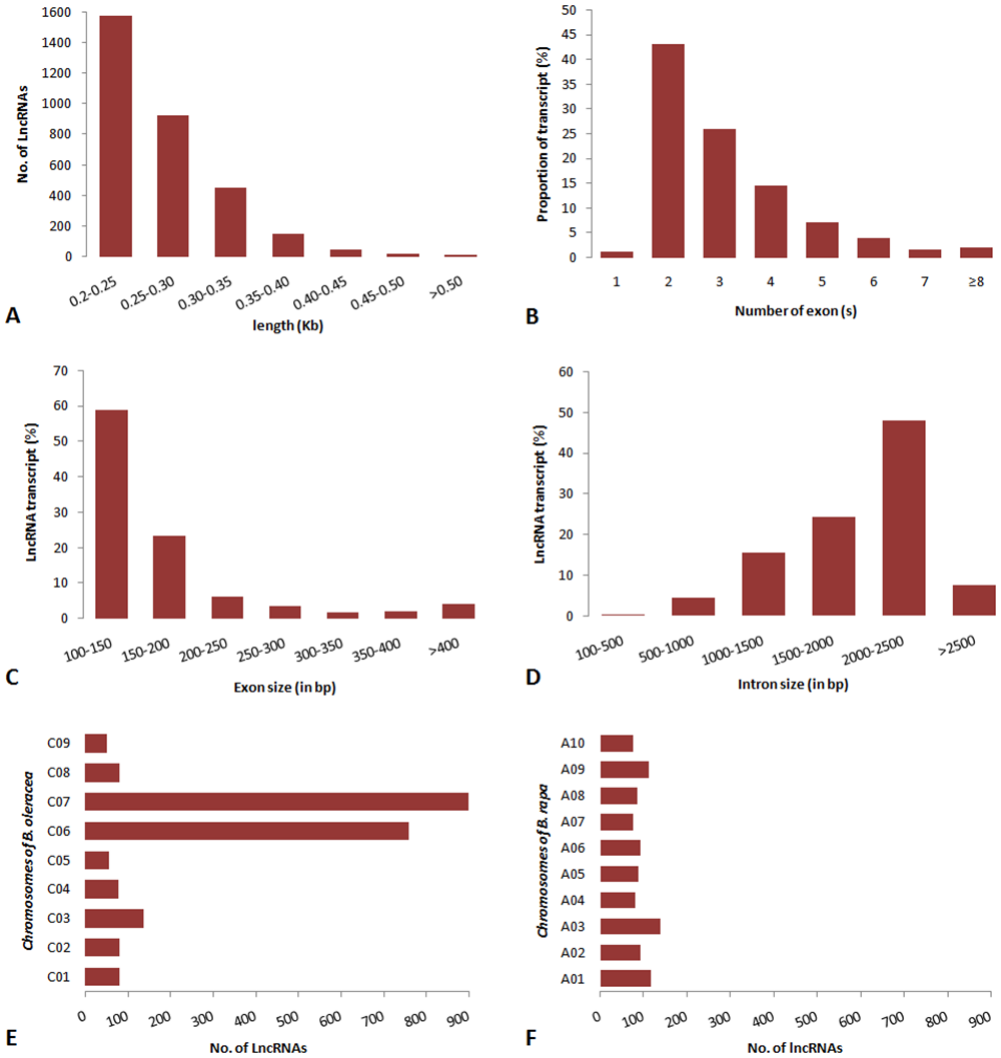
Features and distribution of lncRNA structures in *B*. *napus*. (A) Length distribution of 3,181 *B*. *napus* lncRNAs. (B) Number of exons per transcripts for all lncRNA transcripts. Size distribution of exons (C) and introns (D) for lncRNAs. Number of lncRNAs on each chromosome of the reference genomes of *B*. *oleraceae* (E) and *B*. *rapa* (F).

Based on the above results, 3,181 novel *B*. *napus* lncRNAs were selected for further analysis. The size distribution of these lncRNAs ranged from 200 bases to 5,782 bases, with more than 90% lncRNAs ranging from 200 to 350 bases (Fig 2A). Characterization of the genomic structure revealed that 1.3% of *B*. *napus* lncRNAs have only one exon, 43% have two exons and 26% have three exons while the rest have more than three exons (Fig 2B). From the category of lncRNAs with multiple exons, 67 lncRNAs had more than eight exons. Size distribution of the exons showed that 82% of the lncRNA exons had sizes ranging from 100 to 200 bases (median 146 bases; t-test, P-value ≤ 0.00011) (Fig 2C). Similarly, *B*. *napus* lncRNAs possessed longer intronic regions with sizes ranging from 1500 to 2500 bases (median 2198 bp; t-test, P-value ≤ 0.00037) for 72% of the introns (Fig 2D). Gene structure analysis of human lncRNAs has shown that the lncRNA exons are of similar length to protein coding exons and introns are longer than those of the protein coding transcripts [55]. However, since the majority of human lncRNAs have two to three exons as in case of *B*. *napus* lncRNA in this study, the overall lncRNA transcripts are shorter than protein coding transcripts.

The availability of the AA (*B*. *rapa*) and CC (*B*. *oleracea*) genomes as references provides the possibility to determine the origin of *B*. *napus* lncRNAs and understand their evolution in *B*. *napus*. Among the identified lncRNAs, 2,200 and 961lncRNAs were assigned positions across nine and ten chromosomes of CC *(B*. *oleracea)* and AA (*B*. *rapa*) genomes, respectively. This suggests that *B*. *oleracea* CC genome has contributed a larger number of lncRNAs to *B*. *napus*. Out of 2,220 lncRNAs in *B*. *oleracea*, 1,660 were located only on chromosome 6 (C06) and chromosome 7 (C07) and constituted the majority of stress responsive *B*. *napus* lncRNAs (Fig 2E). The remaining 560 lncRNAs were uniformly distributed amongst other chromosomes. Chromosome C09 (containing only 50 lncRNA loci) had the lowest lncRNA density. In the *B*. *rapa* genome, chromosome 3 (A03) had the highest number of lncRNA loci (138), followed by A01 (116), A09 (112) and A06 (94) (Fig 2F). In addition, 532 lncRNAs were conserved between both the genomes and were evenly distributed throughout the chromosomes suggesting that they are ancient and well-conserved. Moreover, the majority of intergenic lncRNAs were AA or CC genome specific suggesting that *B*. *napus* intergenic lncRNAs are probably more recent and may have evolved after speciation of *B*. *rapa* and *B*. *oleracea* separately or very recently after the speciation of *B*. *napus*. Future investigation into the genomic synteny analysis of the predicted lncRNAs between the AA and CC sub genomes will provide additional insights into the origin and evolution of *B*. *napus* lncRNAs.

### *B*. *napus* lncRNAs differentially expressed in response to *S*. *sclerotiorum*

**Fig 3:**
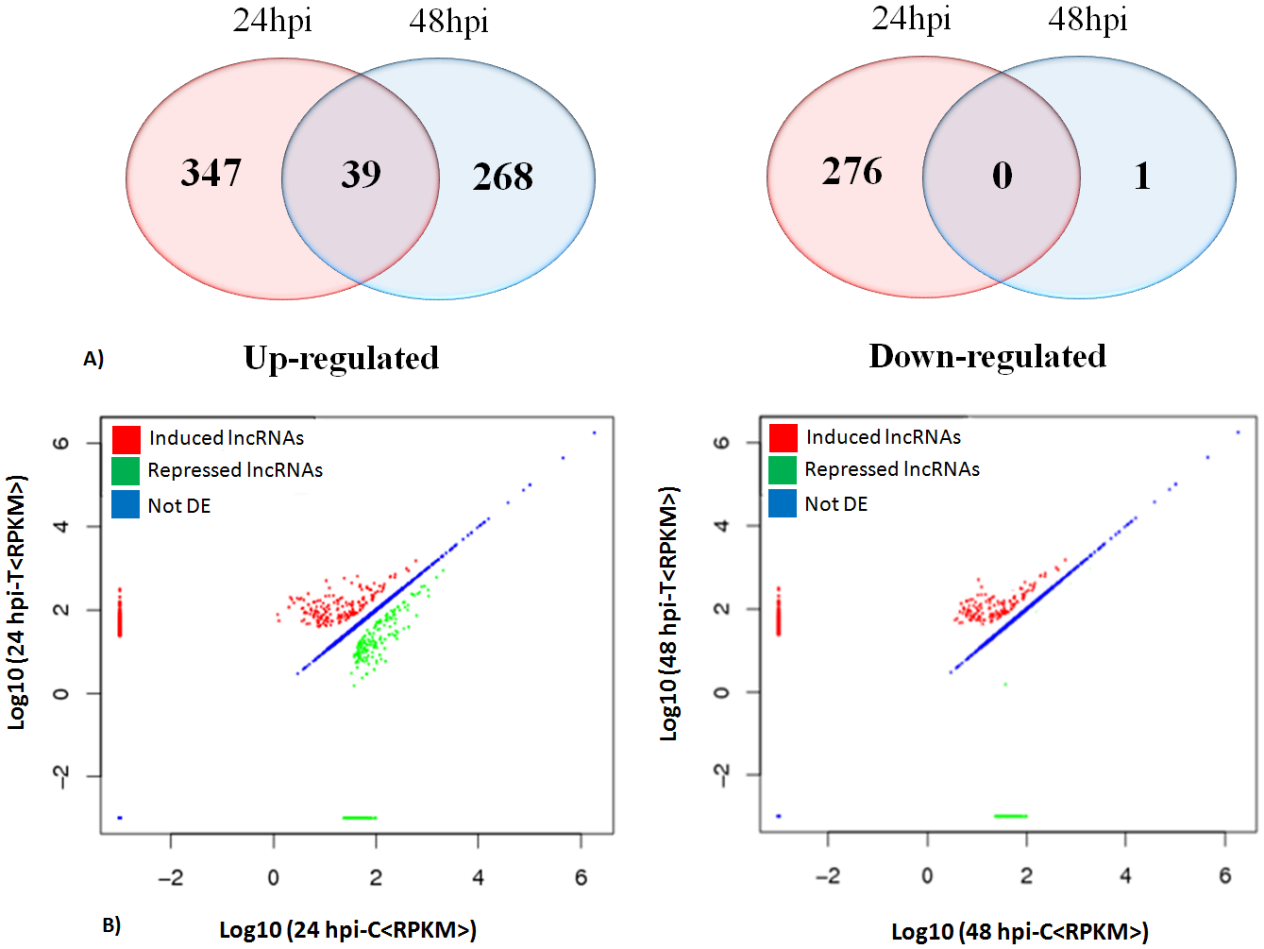
Differential expression of *B*. *napus* lncRNAs post inoculation with *S*. *sclerotiorum*. Venn diagram representation of differentially expressed lncRNAs at 24 hpi (A) and 48 hpi (B). Scatter plot representing the correlation analysis of differentially expressed lncRNAs at 24 hpi (C) and 48 hpi (D).

Recent reports have shown that many lncRNAs actively participate in the regulation of various stress responses including abiotic stresses and pathogen infection [56]. In *Arabidopsis*, 20 *F*. *oxysporum* responsive novel lncRNAs were identified using a strand specific RNA sequencing, including five lncRNAs related to disease development [28]. Similarly, 125 putative lncRNAs responsive to powdery mildew infection and heat stress have been identified in wheat [27]. In the present study, the normalized expression pattern of *B*. *napus* lncRNA reads of control and inoculated samples were calculated using FPKM (fragment per Kilobase of transcript per million mapped reads) and compared at different time points to identify *S*. *sclerotiorum* responsive lncRNAs. Based on the statistical analysis of our results, 931 *B*. *napus* lncRNAs were significantly (P≤ 0.05) differentially expressed in response to *S*. *sclerotiorum* infection. Of these, 386 and 307 lncRNAs were induced in response to the pathogen and 276 and one lncRNA were repressed in the 24hpi and 48hpi samples, respectively (Fig 3A) (S5 Table). We also observed that the abundance of 39 lncRNAs was significantly (P≤ 0.05) increased at both the time points. The number of DE *B*. *napus* lncRNAs was higher at 24 hpi than 48 hpi. This is further supported by the fact that, the correlation of the lncRNA expression levels between 24 hpi control vs.24 hpi pathogen challenged (R = 0.939; P<0.01) were higher when compared 48hpi control vs.48 hpi pathogen challenged (R = 0.82; P<0.01) (Fig 3B). This suggests that *B*. *napus* lncRNA mediated post transcriptional changes might be occurring at 24 hpi of *S*. *sclerotiorum* challenge which gradually decreased by 48 hpi. This could be due to increase in virulence and high necrosis at 48 hpi which might have resulted in very little tissue for isolating lncRNAs.

### Classification of lncRNAs into ncRNA families in *B*. *napus*

To annotate lncRNAs from an evolutionary point of view, we classified the predicted lncRNAs into different ncRNA families using INFERNAL [47]. Based on a consensus secondary structure annotation using a covariance model, we identified 415 unique non-coding sequences belonging to 126 conserved lncRNA families from the libraries at both the time points (S6 Table). Among the conserved lncRNA families, four families (tRNA, snoR71, mir156 and mir169) accounted for more than 10 members. Moreover, several small nucleolar RNA families (SNORD14, SNORD18, SNORD25, snoR71, snoR116) constituted the major categories of lncRNAs in *B*. *napus* with more than 5 members. A recent report on the comparison of the genomic position of human lncRNAs with small RNAs (sRNAs) on the same strand revealed that the lncRNA exons are enriched for all classes of small RNAs with the exception of snRNAs and snoRNAs [55]. A similar study in wheat reported 26 lncRNAs as being putative precursors for 97 siRNAs and snoRNAs involved in resistance to powdery mildew infection [27]. Consistent with other results, it is possible that *B*. *napus* lncRNAs might serve as precursors for different types of functional sRNAs with or without having intrinsic functionality within them.

Additionally, many non-plant specific lncRNA families including the human nuclear enriched abundant transcript 1 conserved region 2 (NEAT1_2), X inactive specific transcript (XIST) and small cajal body-specific RNA 7 (SCARNA7) [56–59], bacterial small transcript non coding 430 (STnc430) and fungal small nucleolar RNA (Fungi_U3) [60] were also represented by one or more lncRNA members in *B*. *napus*. This suggests that, lncRNA transcripts may be evolutionarily conserved across the living systems. As it is difficult to compare divergent nucleic acid sequences, it may be assumed that lncRNAs are relatively conserved as gene units.

### *B*. *napus* lncRNAs as potential miRNA precursors

**Fig 4:**
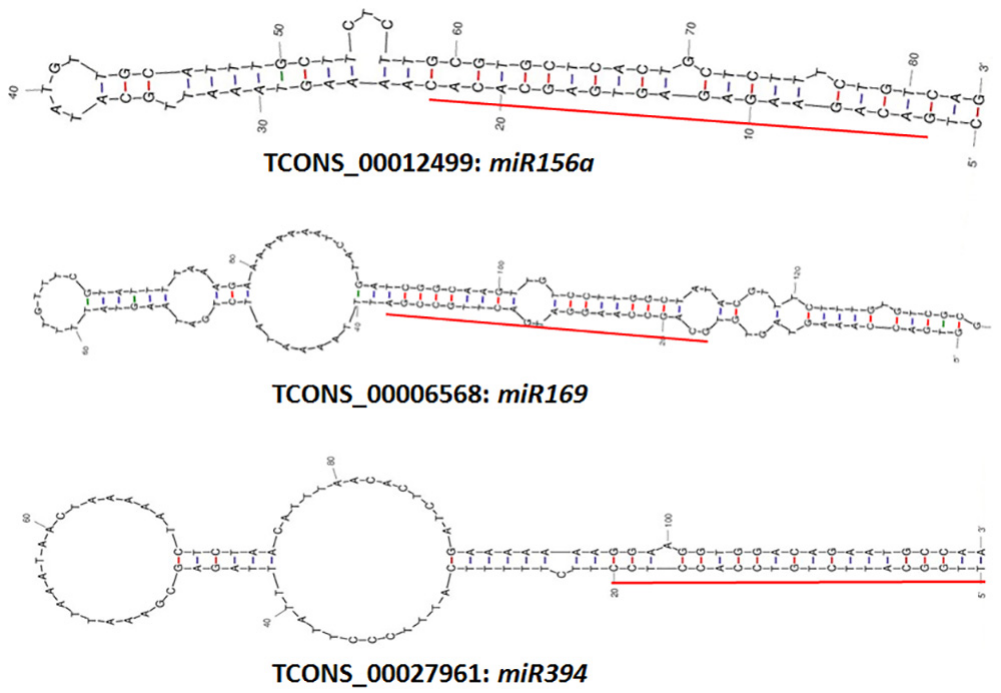
Representative potential miRNA precursor sequences in differentially expressed lncRNAs of *B*. *napus*. The red lines indicate the mature miRNA sequences.

MicroRNAs (miRNAs) are small RNAs of 20-22nt in length and act as an important class of ncRNAs involved in the regulation of gene expression at both transcriptional and post-transcriptional levels in plants [61]. These miRNAs are usually generated via sequential cleavage of long precursor transcripts by Dicer-like enzyme [62]. A single long non-coding transcript can be processed into multiple miRNAs, each with a distinct sub-cellular localization and unique function [5–6]. Since the mature miRNAs target multiple sites on mRNAs resulting in translational repression and gene silencing, identification of miRNAs and their targets could aid in our understanding of regulatory processes involved in mediating stress response in plants. Recent genome-wide studies have revealed that a major fraction of long non-coding transcripts in eukaryotes function as precursors for miRNAs [1, 14, 63]. In human keratinocytes and neonatal mice, miR675 has been derived from the lncRNAH19 and is endogenously expressed [64]. Similarly, four lncRNAs responsive to powdery mildew infection were found to be precursors for miR2004, miR2010 and miR2066 and were simultaneously up-regulated post pathogen challenge [27]. In the present study, alignment of 3,181 *B*. *napus* lncRNAs against the miRBase resulted in the identification of 28 lncRNAs having high homology (>90%) with known miRNA precursors from *B*. *napus*, *B*. *rapa*, *A*. *thaliana* and *A*. *lyrata* (S7 Table). Additionally, miRPara analysis of the lncRNA datasets revealed 13 lncRNAs with precursor sequences for 96 probable novel miRNAs from *B*. *napus*. Five lncRNAs (TCONS_00012499; TCONS_00004577; TCONS_00015411; TCONS_00004034 and TCONS_00009614) served as precursor for miR156, while another five lncRNAs (TCONS_00006568; TCONS_00018692; TCONS_000017152; TCONS_00008591; TCONS_000010926) were precursors for miR169 (Fig 4). Both miR156 and miR169 have been earlier reported to be significantly up-regulated post infection with fungal phytopathogens such as *Dothiorella gregaria* and *Botrytis cinerea* [65–66]. Three lncRNAs (TCONS_00027961; TCONS_00027962; TCONS_00011200) carried the precursor sequence for miR394 (Fig 4), whose potential targets are the F-box family proteins. Furthermore, eleven novel *B*. *napus* miRNAs identified through mirPara analysis also targeted F-box proteins. A recent report has shown that the expression of *Botrytis cinerea* responsive miR394 in *Lycopersicon esculentum* is negatively correlated with that of its target F-box protein [66]. The negative regulation of COI-F-box proteins in host plant is often associated with compromised Jasmonic Acid (JA) signaling and high sensitivity to necrotrophic pathogens [67]. Therefore, it can be suggested that, biosynthesis of miR394 by three *B*. *napus* lncRNAs might be responsible for cleavage of F-box encoding transcripts which in turn may alter the JA dependent defense response against *S*. *sclerotiorum*. However, this suggestion needs to be confirmed through experiments, which are beyond the scope of this current study.

### Analysis of interaction between complementary lncRNA-mRNA in *B*. *napus*

**Fig 5:**
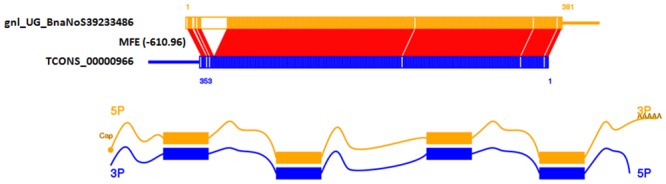
Complementary interaction analysis of a novel up-regulated lncRNA and sense coding transcripts of *B*. *napus* in response to *S*. *sclerotiorum* using RNAplex. “gnl_UG_BnaNoS39233486” is the gene ID of a messager RNA and “TCONS_00000966” is the ID of correspond lncRNA. Minimum free energy (MFE) is shown in the bracket at the centre.

LncRNAs control various aspects of post-transcriptional mRNA processing through complementary base pairing with coding transcripts [68]. This sense-antisense base pairing is critical for the regulation of transcription and stability of the target mRNA [50]. To determine the role of antisense lncRNA in modulating transcription and mRNA stability, we used RNAPlex [50] to perform comparative interaction analysis between *B*. *napus* antisense lncRNA and the corresponding sense mRNA. Out of the 15 antisense lncRNAs identified in the present study, 10 lncRNAs showed more than 60% complementary base pairing with the sense mRNA. Specifically, antisense lncRNA TCONS_00000966 exhibited more than 90% complementary base pairing with the corresponding sense gene *BnaNo39233486*, which encodes for a plant defensin (PDF) protein (Fig 5). Diverse antisense transcript expression was also detected at a region with three defensin like genes in *Arabidopsis*, post inoculation with *F*. *oxysporum* [28]. Plant defensins are small basic peptides similar to those found in animals, produced in plants as part of an innate immune response and exhibit antifungal activity against a broad range of plant pathogens [69]. It can be assumed that TCONS_00000966 might have a significant role in the modulation of *BnaNo39233486* by complementary base pairing during pathogen infection. Therefore, future investigation towards development and characterization of knockout mutants for lncRNA TCONS_00000966 will be useful to determine its functional role in *B*. *napus* defense response to *S*. *sclerotiorum* infection.

### Upstream/downstream lncRNAs regulating *B*. *napus* genes

Transcription from an upstream non-coding promoter can positively or negatively affect expression of the downstream coding gene by inducing chromatin remodelling or by inhibiting RNA polymerase II recruitment [5]. In humans, a ncRNA transcribed from an upstream region of the Dihydrofolate reductase (*DHFR*) locus forms a stable complex with the major promoter of *DHFR* and prevents the binding of the transcriptional co-factor transcription factor II D (TFIID) leading to the repression of *DHFR* gene [70]. Similarly, downstream lncRNA may bind to the 3’ UTRs of the coding sequence and could be involved in intergenic regulatory mechanisms [71]. In the present study, of the 2,821 *B*. *napus* lncRNAs located in the intergenic regions, 500 DE lncRNAs were annotated to the upstream and 512 DE lncRNAs were annotated to 3’ UTR and downstream of the coding transcripts (S8 Table). Specifically, nine lncRNAs from the intergenic regions of *B*. *napus* defense responsive genes were significantly differentially expressed in response to *S*. *sclerotiorum* (Table 2). For instance, lncRNA TCONS_00021690 located in the positive strand upstream of the *B*. *napus* gene, *BnaNoS38899684* encoding F-box protein was found repressed (-1.3 fold at 24 hpi;-0.59 fold at 48 hpi) in response to *S*. *sclerotiorum* infection. Similarly, lncRNA TCONS_00021619 located in the upstream of the *B*. *napus* gene, *BnaNoS55191345* encoding *Lipoxygenase 4* was significantly induced (6.69 fold at 24 hpi; 14.48 fold at 48 hpi) post infection with *S*. *sclerotiorum*. Our results suggest that *B*. *napus* lncRNAs in the intergenic regions might overlap with the cis-regulatory elements of the coding transcripts but exhibit contrasting roles towards transcriptional regulation of the defense responsive genes. Further research on the specific role(s) of these lncRNAs will provide additional information about their detailed roles in disease development or pathogen defense.

**Table 2:** List of selected DE lncRNAs located upstream or downstream of defense responsive genes of *B*. *napus*.

LncRNA ID	Chr	Bracketing gene	Up/down stream	Bracketing gene annotation	Expression of gene
TCONS_00003548	A03	BnaNoS37273850	UPSTREAM_2K	Ehylene-responsive element binding factor 5	Induced
TCONS_00021619	C06	BnaNoS55191345	UPSTREAM_2K	Lipoxygenase 4	Induced
TCONS_00027151	C07	BnaNoS53250997	UPSTREAM_2K	Allene oxidase cyclase	Induced
TCONS_00021690	C06	BnaNoS38899684	UPSTREAM_2K	F-box protein	Repressed
TCONS_00018913	C03	BnaNoS31705296	UPSTREAM_2K	Glutathione S-transferase TAU 12	Induced
TCONS_00012312	A09	BnaNoS52591963	DOWNSTREAM_2K	Glucan endo-1,3-beta-glucosidase 10	Repressed
TCONS_00026803	C07	BnaNoS39174956	DOWNSTREAM_2K	Transcription factor subunit NF-YB3A	Repressed
TCONS_00026707	C07	BnaNoS25441206	DOWNSTREAM_2K	Glycine decarboxylase P-protein	Repressed
TCONS_00013960	A10	BnaNoS39270037	DOWNSTREAM_2K	Tetratricopeptide repeat domain protein	Repressed

### Validation of lncRNA expression using qRT-PCR

**Fig 6:**
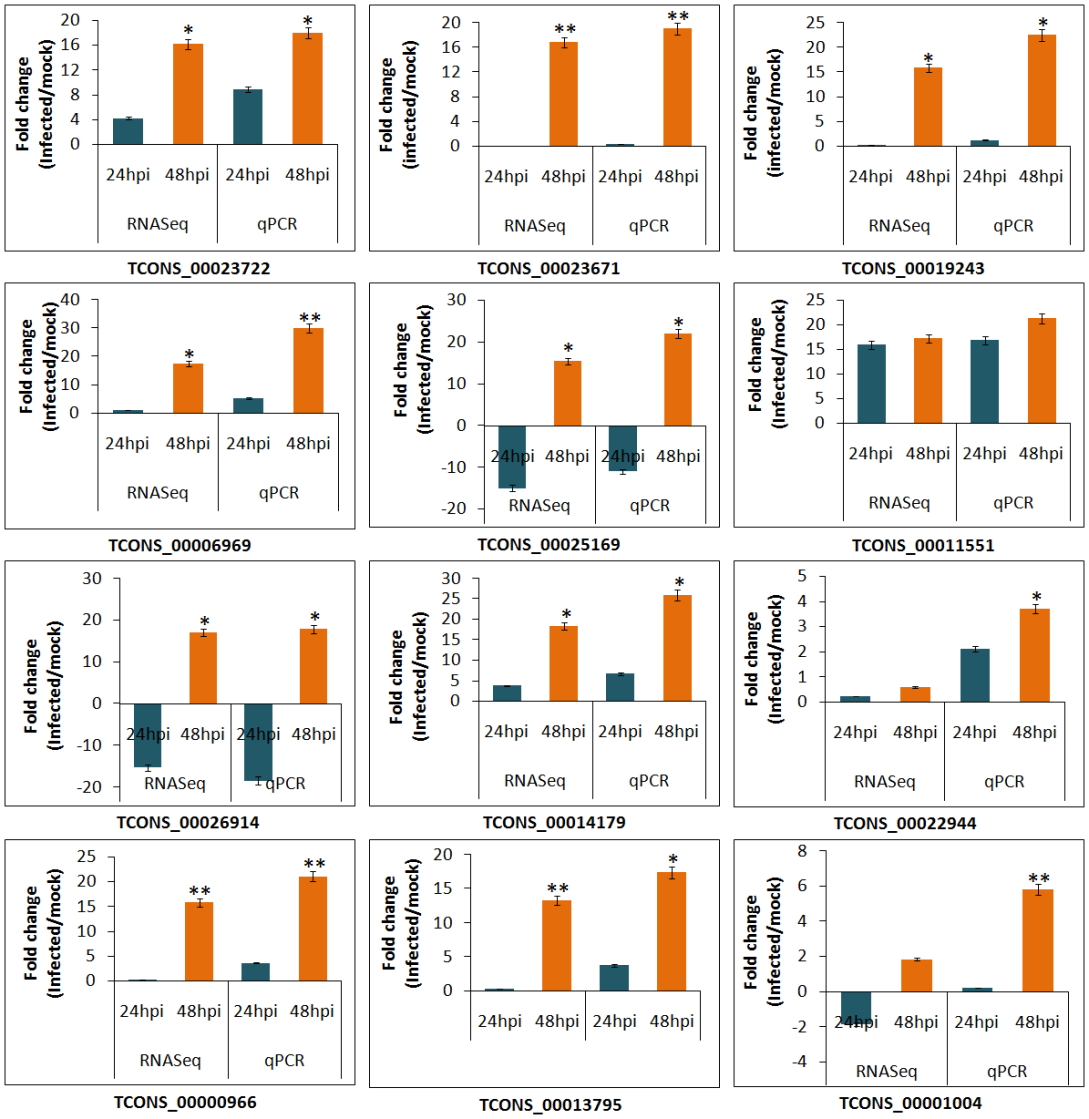
qRT-PCR validation of pathogen induced fold changes in 12 selected *B*. *napu*s lncRNAs detected by RNA-seq. At each time point, the expression level in *S*. *sclerotiorum* infected sample was normalized to that in its corresponding mock sample. Standard error bars for the fold changes determined by qRT-PCR are shown.

To validate the lncRNA expression results obtained from the RNA-Seq experiment, we used qRT-PCR to investigate the expression of *B*. *napus* lncRNAs (TCONS_00023722, TCONS_00023671, TCONS_00019243, TCONS_00006969, TCONS_00025169, TCONS_00011551, TCONS_00026914, TCONS_00014179, TCONS_00022944, TCONS_00000966, TCONS_00013795 and TCONS_00001004) with a range of expression levels in response to *S*. *sclerotiorum*. As shown in Fig 6, the expression patterns of the pathogen responsive lncRNAs as investigated by RNA-Seq and qRT-PCR were relatively consistent with similar trends. However, the relative expression levels of all lncRNAs as evaluated by qRT-PCR were slightly higher than indicated by RNA-Seq. For example, *B*. *napus* lncRNA TCONS_00014179 showed an increased abundance of 3.74 fold at 24 hpi and 18.37 fold at 48 hpi with RNA-Seq while the observed increase in abundance was 6.65 fold at 24 hpi and 25.92 fold at 48 hpi with qRT-PCR. This difference in transcript abundance can be attributed to differential sensitivity of RNA-Seq and qRT-PCR towards normalization of gene expression data [72]. Thus, the validation of the RNA-Seq results by qRT-PCR supported the fact that the size normalized expression values (RPKM) obtained with RNA-Seq data can be efficiently used to determine the lncRNA transcript expression, and further confirmed a significant role for these lncRNAs in *B*. *napus-S*. *sclerotiorum* interaction.

### Verification of sense/antisense transcript expression in *B*. *napus*

**Fig 7:**
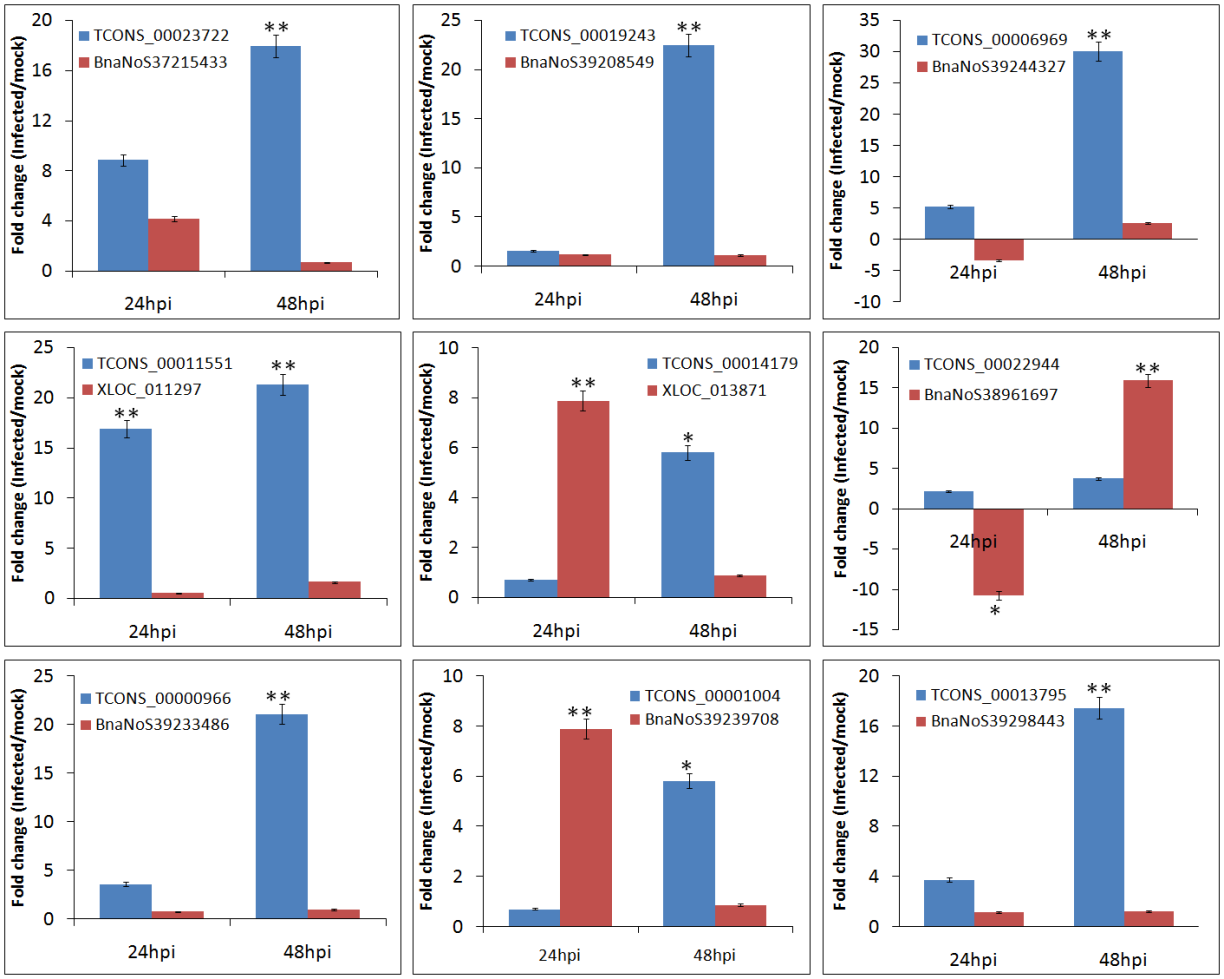
qRT-PCR verification of selected lncRNAs and the corresponding sense coding transcripts from *B*. *napus* detected by RNA-Seq. At each time point, the expression level in *S*. *sclerotiorum* infected sample was normalized to that in its corresponding mock sample. Two internal controls (*Actin* and *UBC9*) were used for data normalization.

Antisense lncRNAs exhibit different modes of actions including inhibition of the synthesis of sense transcript, modulation of RNA splicing and controlling the abundance of sense RNA [73]. In the present study, nine pairs of the sense and antisense transcripts were selected for further verification using qRT-PCR to determine the involvement of the antisense lncRNAs on the expression of coding genes in *B*. *napus* (Fig 7). For example, antisense lncRNA TCONS_00001004 revealed low transcript abundance (0.703 fold) at 24 hpi and significant induction (7.89 fold) at 48 hpi while the corresponding *B*. *napus* sense transcript BnaNoS39239708 showed higher transcript abundance (5.81 fold) at 24 hpi and lower expression (0.875) at 48 hpi. Similar results were also observed for six pairs of sense/antisense transcripts in *Neurospora crasa* [73]. The influence of antisense transcript on the expression of sense gene largely depends upon the nature of overlap between them. The expression levels of sense transcript have been reported to decrease significantly with increase in overlap between the sense and the antisense pairs in human and mouse [74]. This is in accordance with the hypothesis that steric clashes of transcriptional machinery through collision between RNA polymerase II leads to depleted expression levels of the transcripts [75]. Further, our study also revealed that the other eight *B*. *napus* antisense transcripts with significant expression post infection with *S*. *sclerotiorum* had no associated expression of the sense transcripts (data not shown). This may be due to the absence of sense transcriptional activators or presence of strong antisense promoters resulting in steric clashes of the transcriptional machinery and subsequently leading to the suppression or termination of transcription from the sense promoter.

## Conclusions

By applying a strand specific approach we have systematically identified, for the first time, 3,181 novel lncRNAs in *B*. *napus*. Moreover, our study revealed 931 *B*. *napus* lncRNAs responsive to *S*. *sclerotiorum* infection suggesting that they play important roles in mediating host responses to the pathogen. The lncRNAs were classified into 126 families with broad conservation across eukaryotes and some of them appeared to serve as precursors for major stress responsive miRNAs. Additionally, we also identified many lncRNAs overlapping with the cis-regulatory regions of *B*. *napus* DEGs with possible role in defense response, which needs further elucidation. Overall, our study has demonstrated that lncRNAs may be important in mediating responses of *B*. *napus* to *S*. *sclerotiorum* and will provide a starting point for future investigation into the molecular mechanisms and regulatory functions of lncRNAs.

## Supporting Information

S1 Table List of primers used for qRT-PCR. (XLSX)

S2 Table Map summary of the RNA-Seq reads from 12 stranded *B*. *napus* libraries post infection with *S*. *sclerotiorum*. (XLSX)

S3 Table List of 17,964 un-annotated transcripts obtained through initial assembly with Cufflinks. (XLSX)

S4 Table List of 3,181 novel *B*. *napus* lncRNAs. (XLSX)

S5 Table List of differentially expressed *B*. *napus* lncRNAs post inoculation with *S*. *sclerotiorum*. (XLSX)

S6 Table Classification of predicted lncRNAs into different non coding RNA families. (XLSX)

S7 Table *B*. *napus* lncRNAs serving as potential miRNA precursors. (XLSX)

S8 Table *B*. *napus* differentially expressed lncRNAs located upstream or downstream of coding transcripts. (XLSX)
